# Toward an equitable transportation electrification plan: Measuring public electric vehicle charging station access disparities in Austin, Texas

**DOI:** 10.1371/journal.pone.0309302

**Published:** 2024-09-05

**Authors:** Junfeng Jiao, Seung Jun Choi, Chris Nguyen

**Affiliations:** 1 Urban Information Lab, School of Architecture, The University of Texas at Austin, Austin, Texas, United States of America; 2 Transportation and Public Works Department, Active Transportation and Street Design, City of Austin, Austin, Texas, United States of America; Joginpally B R Engineering College, INDIA

## Abstract

The deployment of public electric vehicle charging stations (EVCS) is a critical component of transportation electrification. Recent studies have highlighted growing concerns about disparities in accessibility to public chargers between different demographic groups. This research expands ongoing equity concerns by contextualizing existing transportation equity discourse and analyzing public charger access disparities in Austin, Texas. Using threshold equity toolkits, we investigated public EVCS access disparity across different races and income groups. We conducted a generalized additive model regression to measure and visualize the effects of possible determinants on public EVCS access. The analysis results revealed that a public EVCS access disparity exists in Austin, with most chargers being installed in areas where the majority of the population is Non-Hispanic White. There was a more equal distribution of public EVCSs across income quartiles when compared with race. However, middle- and high-income groups had better access than lower-income communities in terms of distance to the nearest public EVCSs. Our regression analysis found that regional and socio-demographic factors, such as race and income, have a statistically significant impact on public charger access. The regression analysis also revealed that Austin’s current public EVCS deployment seems to favor communities above the poverty level and with higher numbers of registered electric vehicles. Local policymakers should reflect on the findings of this study to develop an equitable transportation electrification plan. Federal environmental justice plans such as the Justice40 initiative can benefit from incorporating more local contexts to better invest in disadvantaged communities.

## 1. Introduction

Transportation electrification is essential for achieving climate goals and promoting low and zero carbon emissions in urban mobility systems. To achieve this, the government has introduced legislation and incentives to promote the shift to Electric Vehicles (EVs) nationwide. In December 2021, the U.S. Secretary of Energy signed a memorandum to launch the Joint Office of Energy and Transportation, which aims to allocate $7.5 billion from the Infrastructure Investment and Jobs Act to build a national electric vehicle charging network [1].

To facilitate effective transportation electrification, local governments are encouraged to provide incentives to promote the purchase of EVs and installation of at-home and public Electric Vehicle Charging Stations (EVCSs). However, there is a possibility that EV purchases and EVCS installations are not equitable. Several studies indicated that EVs are considered to be luxury vehicles and their adoption is skewed towards those who have a higher income and higher education levels, who are likely to be single-family homeowners [2–4]. EV buyers also tend to be male and own multiple vehicles [5]. EV purchase rebates have been predominantly given to these high-income buyers, mostly in major metropolitan regions [6]. Conversely, lower income, black-dominant, and disinvested neighborhoods are less likely to have EV chargers deployed than affluent neighborhoods [7].

Even with a perfectly equitable distribution of public EVCSs, at-home charging is often more cost-effective than charging at EVCS. At-home charging is often done at night, which is priced lower when using off-peak electricity rates [8]. According to the U.S. Department of Energy [9], fully charging a 100-mile range EV costs between ¢8 and ¢27 per kWh on average at home but can cost ¢31–43 per kWh at a public charging station [5, 10]. Single-family households are better able to install personal chargers than residents of multi-family dwellings [11]. The affordability of purchasing and installing an at-home charger and access to different levels of existing charging stations can influence an individual’s decision to purchase an EV. The cost of owning an EV can extend beyond the capital cost of installing a home charger and includes the ability to recover those costs over time [11].

EVCSs are a critical component for building a robust transportation electrification system, but the benefits of transportation electrification may not be shared equitably. The definition of what is equitable remains subject to debate. Multiple studies have introduced distributional philosophies into the discourse on equity [12]. Carlton and Sultana [12] coined the term ‘charging equity’ to describe the field that examines equity issues within EVCSs. They describe this field as one that applies distributional approaches, emphasizing disparities in access and behaviors related to charging resources between high and low EV adoption groups. Lower adoption groups can be identified as ’disadvantaged’ in planning history. The discourse on ’charging equity’ forms part of the broader conversation on transportation equity. A study in California by Hsu and Fingerman [13] revealed that Black and Hispanic populations and below-median income geographic units have less access to public EVCSs. Their study also mentions that EV infrastructure was likely deployed in this manner to facilitate EV adoption, which could lead to inequitable outcomes. Existing charging equity literature confirmed their findings in California [3, 14], Chicago [15], NYC [7], and Seattle [16] in North America.

The primary objective of this article is to bridge the knowledge gaps by: 1) diversifying the case studies to include regions with increasing EV adoption that have not been extensively examined; 2) enriching the discourse with more explicit studies on charging equity to mitigate the continued disparities; and 3) bringing a holistic approach to charging equity in the context of the broader transportation equity literature.

Despite the growing body of research, charging equity studies likely exhibit a bias towards the West Coast. Hopkins et al. [17] analyzed seven sets of literature on this topic in North America; five were based on the West Coast (California, Seattle, and Los Angeles), while only two were from the East (Chicago and NYC). It’s noteworthy that states like Florida and Texas, despite being leaders in EV adoption in North America after California [18], have not been included in such studies. Moreover, there is a scarcity of studies in charging equity literature that ’explicitly’ incorporate equity language and normative arguments [12]. Out of 37 peer-reviewed articles in mainstream journals, only 29.7% explicitly address equity in the distribution of charging facilities for EVs [12]. The prevailing studies embrace a utilitarian perspective, prioritizing the overall utility of installing and operating chargers, often neglecting distributional equity [12]. Hence, the literature on explicit charging equity remains fragmented and inconsistent, with the equity focus of some studies often overlooked by others.

We conducted a transportation equity analysis on public EVCS access in the Austin Metropolitan Area of Texas (TX). Our research question centers on testing the hypothesis of whether distributional charging equity issues are present in Austin, TX. Austin stands out as a focal city in EV adoption in the Southern U.S., as evidenced by having the highest ratio of registered EVs in TX. As of December 27, 2022, Austin had the third-highest number of EV registrations after Dallas-Fort Worth and Houston. 21% of all EVs in Texas are registered in Austin. The ratio of registered EVs to total vehicle registration is highest in Austin at 1.71% [19]. Although the ratio seems small, the city has set ambitious goals for transportation electrification. According to the Austin Climate Equity Plan [20], the city aims to have 40% of vehicles in Austin be electric by 2030. Austin has a history of racial segregation, with pronounced disparities emerging following the construction of Interstate Highway 35 (I-35). Considering these historical inequities, the city has prioritized equity initiatives in addressing contemporary urban challenges. The city’s proactive efforts to promote equity in transportation electrification planning, done against the backdrop of racial disparity issues seen in many other U.S. cities, make it an ideal model for other cities in North America. For instance, in 2023, the city launched the Equitable Transit-Oriented Development Plan. One key aspect of this plan highlighted the availability of EVCS as a key element in ensuring equitable access options and called for further discussion.

The remainder of this article is structured as follows. First, we present an overview of existing local and federal plans and policy initiatives, including a review of literature on transportation equity. Next, we explain the materials and methods used in the study, including the equity toolkits we employed. Then, we present the findings of our study in detail. Lastly, we discuss the limitations of our study and suggest future steps for further research.

## 2. Literature review

### 2.1. Level of services and current policy initiatives

Different levels of charging stations can play a crucial role in transportation electrification. There are three levels of EVCS services, ranging from Level 1 to Level 3. Level 1 and Level 2 chargers are composed of plug-in electric sockets with voltages of 110/120V and 220/240V, respectively, and are commonly used for residential charging. Level 3 chargers, also referred to as Direct Current (DC) fast charging, use a higher voltage of 480V and are commonly used for public charging. This is because the installation costs alone cost upwards of $40,000 to $50,000, which is ‘cost-prohibitive’ for residential usage [11]. According to the U.S. Department of Energy [21], the installation cost ranges of Levels 1 to 3 are $300 to $15,000, $400 to $6,500, and $10,000 to $40,000, respectively. However, higher levels of EVCS may have other costs, including permitting, electrician assessments, electrical panel upgrades, and other modifications to existing constructions that can greatly increase the total cost of installing a charger. For example, Level 2 commercial sites that require special trenching or boring would cost 25% more than those that don’t require any additional work [11].

In Europe, the EU launched the Clean Energy for All Europeans package in 2019, acknowledging the importance of governance regulation, electricity market design (e.g., tariff and time of use), and adjusting building codes. Complying with the Clean Energy for All Europeans package, smart charging, the distribution of smart meters, and smart pricing play a responsive role [22]. It seems they consider network capacity to be the main obstacle to installing EV chargers.

In the UK, the Automated and Electric Vehicles Act was passed in 2018, giving the government authority to mandate that EV chargers function ’smart’ and meet minimum device-level requirements [23]. Smart charging refers to shifting charging to a different time of day, such as overnight when there is lower demand on the electricity system, rather than during peak times such as 8 a.m. to 11 a.m. and 4 p.m. to 10 p.m. on weekdays [23]. It requires installing a smart chargepoint that is communication-enabled and can automatically respond to remote signals by adjusting the electricity consumption flowing through it [23]. To install a personal chargepoint, you need a Wi-Fi connection, ownership of the property or permission from the landlord, and off-street parking. The cost of a charging point ranges from around £600 to £1000. An Office for Zero Emission Vehicles-authorized installer would need to provide a quote. Distribution Network Operators must approve the installation to help manage grid capacity in the area. This approval process can take considerable time (e.g., 45 days or more), usually making it impossible to install a charger quickly. Once approved, the installation may take up to 90 days from the date of application approval [24].

Additionally, common types of chargepoints differ across the nation, making it difficult to apply the smart charging mechanism uniformly. Considering different governance, regulations, and charging infrastructure, the situation becomes more complex. Tesla’s CHAdeMO is commonly used in the U.S., Europe, and Asia, but specific preferences vary: SAE J1772 is preferred in the U.S., Mennekes in Europe, and BG/T in Asia [25]. Among Asian countries, China and Japan have their common chargers, but South Korea is less significant in this area of research [25].

Public EVCSs typically refer to Level 2 and 3 chargers. Public Level 2 chargers are more common than Level 3 chargers. Ensuring greater access to these charging stations is essential for EV owners. The management of EVCS is closely tied to existing infrastructure and policy. The Texas Electric Vehicle Infrastructure Plan, approved by the Texas Department of Transportation (TxDOT) on July 8, 2022, recognizes this relationship. The plan was developed in collaboration with various government agencies, including those responsible for energy, environment, wildlife, and transportation, as well as the Public Utility Commission of Texas, local councils, service providers, and advocacy groups in the State of Texas. One of the objectives of this plan is to coordinate the efforts of these groups by establishing a cross-agency team known as the ‘EV Working Group.’

TxDOT facilitates the installation of EVCS by contracting with private sector entities on a competitive basis. These vendors work with property owners, utility service providers, and municipalities to complete the installation while following federal requirements and guidelines. The contracts include five-year operations and maintenance plans dependent on each individual location and provisions for vendors to cease operations if necessary. Currently, most chargers are privately owned, with a small number operated by Austin Energy, a publicly owned utility company for the City of Austin (COA). Current rebate and incentive programs for EVCS installation are summarized in S1 Table.

It should be noted that private sector entities prioritize maximizing profits and minimizing costs. This is a likely cause for early EVCS deployments in regions with high EV registration rates. According to the Austin Climate Equity Plan, COA recognizes this issue and has developed strategies to manage their transportation electrification strategy [20]. This strategy includes partnering with local organizations and government organizations to conduct a community needs assessment. The city will prioritize the needs of disadvantaged communities, including low-income and communities of color, by installing more EVCS in these areas. Other strategies include installing EVCS on publicly owned land, continuing to incentivize vendors, adopting new building codes, and expanding outreach efforts. They will also partner with highway and regional mobility authorities to reduce tolls for EVs in historically disadvantaged areas such as the Eastern Crescent. E-bike and electric car-sharing programs will be advertised to promote alternative forms of electric transportation. Finally, the city will partner with different public entities, such as municipal and county governments, school districts, and transportation agencies like CapMetro, to encourage the electrification of public fleet vehicles. Overall, these strategies should promote public efforts to electrify transportation in Austin.

In 2022, the White House introduced the Justice40 initiative, aiming to direct federal investments towards disadvantaged communities, including climate change, clean energy and energy efficiency, clean transit, affordable and sustainable housing, training and workforce development, and the reduction and remediation of legacy pollution [26]. The initiative emphasizes clean transit, focusing on reducing transportation costs and enhancing accessibility. The Justice40 initiative’s Interim Implementation Guidance specifically outlines the clean transportation category, which encompasses the enhancement of public transportation accessibility, reliability, and options, the provision of clean and high-frequency transportation services, and facilitating access to affordable electric vehicles, charging stations, and purchase programs [27].

Supported by the U.S. Department of Transportation, the Argonne National Laboratory developed the Charging Justice40 Map Tool, which was launched on January 28th, 2022 [28]. This tool classifies corridors into two categories: ’EV corridor ready,’ indicating the presence of a sufficient number of EV charging stations, and ’EV corridor pending,’ suggesting a need for more stations. The identification of disadvantaged communities by the Department of Transportation and the Department of Energy involves a multifaceted approach, combining census tracts, tribal lands, and U.S. territories. The criteria for this classification encompass a wide range of indicators, including travel distance, vehicle availability, walkability, transportation costs, disadvantaged demographics (such as the elderly, uninsured, and disabled populations), housing age, environmental justice considerations (like diesel emissions, cancer risk, traffic proximity, water discharge, proximity to hazardous sites like national priorities list, potential pollution mining source, and treatment, storage, and disposal facilities), air quality (PM 2.5 and Ozone levels), access to parks, educational attainment, housing tenure, employment rates, income inequality (Gini index), poverty levels, housing costs, employment in fossil energy sectors, access to utilities, food security, linguistic isolation, homelessness, energy burden, and resilience to climate-related disruptions.

### 2.2. Transportation equity

Transportation equity is a key concern in transportation electrification initiatives. However, the precise definition of what constitutes equity can be challenging. Although the COA has made efforts to promote equity, the details of how they define and measure access disparities are unclear. This is likely due to the nature of the EV market as a public-private partnership, which makes it difficult to understand the exact definitions. Merely increasing the number of EVCS does not necessarily mean that the EVCS are accessible to disadvantaged communities [29].

To ensure that equity concerns are not being touted as lip-service [30], it is important for policymakers to clearly define and understand the concept of transportation equity. This study will contextualize some of the city’s definitions of equity with regards to transportation planning and environmental justice. At its core, transportation equity refers to how a transportation system may benefit or burden certain groups and the extent to which these effects are distributed between said groups [31]. This concept is rooted in environmental justice, which raised concerns regarding the disproportionate distribution of environmental benefits and hazards between different communities [32].

Other conceptualizations of justice have been broadly used in other environmental studies. Wenz [33] defined environmental justice as having access to nutritious food, decent medical care, and the capability to live away from environmental hazards. The ’disproportionate distribution’ of these ‘benefits and burdens’ is key to defining justice [34]. The primary concern is often the disproportionate burden on communities of color. Pellow’s framework [35] of environmental justice, as expanded upon by Sze and London [36], emphasizes that justice is not static. Instead, it is important to address the political, social, and economic interactions leading up to the present to fully understand and address environmental justice.

As stated previously, transportation equity is concerned with how certain groups are benefited and burdened by a transportation system. Historically, discriminatory transportation and housing policies have been used to facilitate segregation and disinvestment in communities of color. [37, 38]. As such, low-income and minority communities are often at the forefront of equity discussions. Based on previous findings of disparities in access and EV ownership [5–7, 13], these communities remain at the center of transportation equity discussions for transportation electrification.

This study focuses on understanding access to public charging infrastructure, which is an important factor in determining the willingness of individuals to purchase EVs. There are several ways to assess the accessibility of EVCS. For instance, it is possible to consider population-weighted travel time by any mode of transport to measure accessibility at the country level [39]. For a less macroscopic approach, Hsu and Fingerman [13] narrowed down the geographic unit of analysis to block groups within a state and measured the nearest distance to highways and the probability of having public EVCSs. In more microscopic analyses, the definition of accessibility may vary based on individual drivers. The simplest approach is the opportunity-based approach, which defines accessibility as the number of charging stations in a threshold range for each household in a community [40].

Defining ’equitable’ access to EVCS typically involves distributional philosophies, addressing social equity and the dispersion of benefits and costs [12]. Equity in EV charging, or ’charging equity’, often adopts a ’simple equality’ approach, discouraging preferential treatment for certain demographic groups and focusing on the needs of historically disadvantaged communities [12]. Several studies have introduced prioritization frameworks aimed at addressing the needs of disadvantaged groups more effectively [13, 41, 42].

Studies suggest that ensuring equal access to chargers is linked to enhancing the ’capability’ to operate EVs, encompassing both the capital costs and the ability to offset these costs [11]. The capital costs cover installation expenses, permits, electrician assessments, electrical panel upgrades, and the installation of electricity meters. The capacity to recuperate these expenses is influenced by factors such as home ownership status [2–4], charging speed, the use of public or private parking, and amenities close to the charging stations [43].

Pereira et al. [44] argue that the capability approach is more comprehensive than simple equality, as it encompasses not only access but also individuals’ opportunities and freedoms. They emphasize the need to define the minimum level of accessibility for basic needs and understand personal abilities to address challenges in distributional charging equity studies. However, the authors highlight two critical challenges in the capability approach: 1) determining the minimum level of accessibility that satisfies an individual’s basic needs, and 2) the necessity of a multidimensional concept to comprehend an individual’s abilities. Addressing these two fundamental challenges is essential for the capability approach to tackle gray areas effectively in distributional charging equity studies.

Studies focusing on charging equity, which explicitly mentions ’equity’ or ’justice,’ tend to favor distributional philosophies. However, the dominant research paradigm in charging equity remains utilitarian social welfare maximization, primarily offering only empirical results [12]. The results of equity analysis vary depending on how we conceptualize equity, whether based on accessibility inequality or accessibility poverty [45]. The application of geographic information systems (GIS), the selection of indices to measure equity, and the use of graduated scales also produce different equity analysis results [46]. The optimum level of analysis varies depending on the scale of the unit of analysis [14]. The density of EVCS placement and the inequity indices showed varying levels of equity [14]. Utilitarianism emphasizes optimizing end-state resource distribution, where equality implies that each individual receives equal consideration in the computation of aggregated social welfare [47]. Typically, the utilitarian approach does not account for distributional differences between groups [48]. There is a distinction between distributional equity philosophies and utilitarian equity philosophies in discussions of charging equity [12].

The current disparities in EVCS access may result from the leading allocation strategies for EVCS. Factors such as traffic volume, parking demand [43], population density, land use, road networks [49], electrical utilities, and management systems [50] are major considerations in conducting suitability analyses for installing chargers. These elements are crucial for the installation of new chargers or for optimization purposes. However, the ’equity layer’ is often overlooked in favor of the utilitarian approach to welfare maximization [12]. It is important to distinguish between studies that validate already installed chargers and studies aiming to optimize the location of new chargers.

### 2.3 Measuring transportation equity

Measuring transportation equity can be a contentious topic, as the definition of equity and how to measure it may not be adequately defined [30]. An approach commonly uses set geographic units with static boundaries to define thresholds based on race or income [51]. However, this approach may reinforce structural disparities and neglect communities that may not fit neatly within defined boundaries [31, 51].

Alternatively, some scholars advocate for a transportation justice approach that does not rely on threshold analysis. Instead, transportation justice should go beyond redistributing outcomes and attempt to consider areas of concern as identified by the communities in question [46]. Justice should integrate practice, context, and engagement [52]. For example, some equity studies directly interviewed the structurally disadvantaged, like LGBTQ+ riders [53]. Karner et al. [31] argued that integrating a society-centric approach into top-down state efforts and reinforcing grassroots community organizing is key to initiating transportation justice. Sheller [52] observes that mobility justice is not static but rather is contextualized through ongoing debates. This perspective is influenced by Dewey’s process of inquiry, which views science as a continuous process where norms and actions mutually influence each other.

However, this study is limited to using an equity threshold toolkit to measure EVCS access disparity due to similar limitations mentioned by Bai and Jiao [54]. First, the public-private nature of the EV market has led to a lack of publicly available data. Second, while the cost burden has been identified, the distribution of static opportunities within Austin has yet to be fully explored. By identifying these disparities, this study aims to provide a foundation for further discussions on equity and justice in transportation electrification.

Our study aligns with the explicit focus on charging equity through a simple equality approach [12]. There is a notable scarcity of multiple case studies that thoroughly address issues related to charging equity. Compared to utilitarian social welfare, a distributional philosophical approach more effectively contextualizes the needs of the disadvantaged. The capability approach requires additional refinement to be practically applicable in analyzing charging equity.

## 3. Materials and methods

### 3.1. Study area

**Fig 1:**
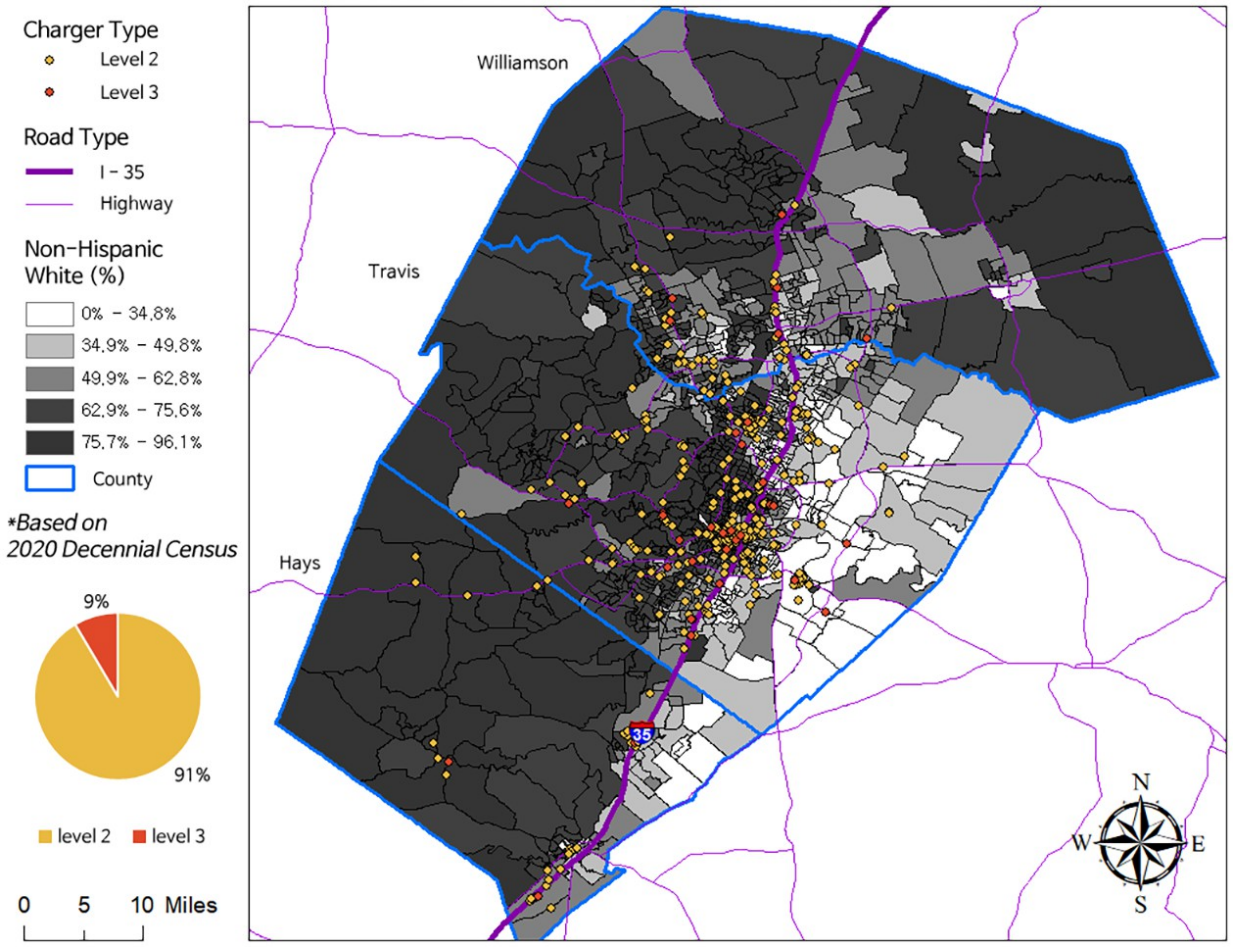
Study area and public EVCSs in Austin, TX.

This study examines the deployment of public EVCSs in Travis, Hays, and Williamson counties. Previous transportation equity analyses in Austin typically focused on Travis County or areas within Austin’s city limits [54]. Our study broadens the scope to three counties that encompass the Austin Metropolitan Area along the I-35 corridor for a more comprehensive understanding of the greater metropolitan area in relation to Austin. Public EVCS data was acquired from the National Renewable Energy Laboratory’s (NREL) alternative fuel station database. This database includes the locations and specifications of all alternative fuel stations in the U.S. and includes both publicly and privately operated EVCS. We utilized NREL’s developer API, which provides a list of public EVCS with location and operating data. This allowed us to filter and select charging stations located within a designated region. When filtering the NREL dataset for the study area, 681 EVCS were identified in the study area. Of these, 623 were Level 2 EVCSs, and 58 were Level 3 EVCSs. As shown in Fig 1, most chargers are in Travis County.

It should be noted that I-35 demarcates a racial barrier between White and minority neighborhoods, as shown in the distribution of Non-Hispanic White populations between Census Block Groups (CBGs). The Justice40 Map Tool highlights that disadvantaged communities are predominantly located in East Austin. This tool’s assessment has highlighted EVCS needs on the I-35 highway corridor. As of June 19, 2023, the tool only includes non-Tesla DC fast chargers. An element visible in the Justice40 Map Tool is missing in Austin Energy, or vice versa.

### 3.2. Variables

Our study calculated the proportion of public EVCSs in a particular CBG relative to all the CBGs in the study area, which is later used as a dependent variable. The dependent variable was defined as the opportunity to access public EVCSs. We calculated the nearest roadway distance from CBG to the nearest public EVCS and the nearest distance from CBG to highways. This was performed by using street network analysis in ArcGIS Pro with 2022 OpenStreetMap data. We used a network distance tool in ArcGIS Pro to find the shortest route along transportation routes. The distance to the nearest EVCS and multi-family proportion were also considered as the control variables.

Addressing factors such as race, education, income, and housing status is crucial for tackling equity issues in charging infrastructure. The lack of adequate transportation access often stems from racial discrimination in housing policies, leading to residential segregation. This, coupled with low-income conditions, such as higher unemployment rates among black communities, contributes to a spatial mismatch. This mismatch exacerbates the unemployment rates in disadvantaged communities by necessitating longer commutes for residents [55]. Furthermore, the link between education and transportation is significant, as access to transportation directly impacts the choices related to schools and travel associated with education. This encompasses aspects such as the mode of transportation, its cost, frequency, speed, and travel distance [56].

We considered household income, poverty level, owner occupancy, education, ownership of a vehicle, and race as independent variables [2–4, 6, 7, 14]. They were obtained from the 2020 Decennial Census and American Community Survey 5-Year (2016–2020) estimates. Household income refers to the median household income. Poverty levels were defined as the proportion of residents above the poverty income level. Owner occupancy was defined as the proportion of owner-occupied housing units compared to the total number of housing units. The education factor was defined as the proportion of residents with bachelor’s degrees or graduate degrees. Ownership of a vehicle was defined as the proportion of residents that own at least one vehicle in the household. More notably, race was considered since many previous studies indicated that there were racial disparities in EVCS access [7, 12]. Race was defined as the proportion of Non-Hispanic Whites in each CBG.

It was also important to address disparities between urban and rural areas. Guo et al. [6] asserted that there were disparities between those living in metropolitan regions and those who did not. Our study added a dummy variable, which defined CBG in urban and rural areas based on the density of housing units. According to the U.S. Census Bureau [57], a CBG with more than 200 housing units per square mile is defined as an urban area. This dummy variable allows us to account for the varying impacts and accessibility in urban versus rural settings.

It should be noted that the EV registration data obtained from Dallas-Fort Worth Clean Cities (DFWCC) [20] was provided at the ZIP code level. DFWCC obtains data from the State of Texas Department of Motor Vehicles registration data and utilizes the Atlas EV Hub Vehicle Identification Number decoder. We areal interpolated the data from the ZIP code level to the CBG level in ArcGIS Pro. Some may question the modifiable areal unit problem when rearranging the spatial scale of vector data [14, 58]. However, both using raw data and aggregating data involve a certain degree of *ecological fallacy* [58]. Social science heavily relies on triple hermeneutics, where the subjectivity of the researcher influences the framing of the hypothesis, the objectives of the research, and the interpretation of the results [59]. Based on pragmatism, scientific inquiry in social science can be justified if the objective is to mitigate the problem with warranted assertibility [60]. Remaining static is worse than attempting to address social issues. Our research problem concerns the disproportionate access to EVCS among different social classes. Census blocks are the smallest unit where census data is provided, followed by CBGs. Rearranging ZIP codes to the smallest unit might be problematic, and using census tracts may result in small sample sizes. Therefore, we chose CBGs as the geographic unit of analysis. This choice balances the need for detailed spatial resolution with the practicality of having sufficient sample sizes to make meaningful inferences.

Areal interpolation involves a five-step process. First, two different shapefiles were imported, and the size of the boundary for each was calculated, ensuring they were based on the same coordinate system. Second, a unique ID was assigned, and the size information was recorded in separate columns for each shapefile, followed by the intersection of the two shapefiles. Third, the area ratio was computed by dividing the area of the intersected shapefile by the area of the corresponding shapefile (in this case, it would be the ZIP code). Fourth, the EV registration record from the previous ZIP code was multiplied by the calculated area ratio (this method preserves the integrity of the CBG). Finally, the intersected shapefiles were dissolved based on the unique ID of the CBG, with records being grouped and summed. The summary statistics of these collected variables can be found in Table 1.

**Table 1:** Summary statistics.

Variables		year	unit	mean	std	min	median	max
**DV**	Public EVCS (Level 2–3)	2022	count	1	2	0	0	27
**IV**	Urban Dummy	2020	(1, 0)	1	0	0	1	1
	Non-Hispanic White	2020	%	0.52	0.21	0.00	0.54	0.96
	Number of Registered EV	2022	count	19	19	0	16	432
	Median Household Income	2020	$	92,441	42,957	2,499	87,874	250,001
	Residents above Poverty Income Level	2020	%	0.89	0.14	0.00	0.94	1.00
	Nearest Distance to public EVCS	2022	meter	3,895	4,774	34	2,312	33,649
	Nearest Distance to Highways	2022	meter	4,866	5,783	0	2,890	33,989
	Multi-Family Housing Units	2020	%	0.30	0.34	0.00	0.15	1.00
	Residents with Bachelor’s degree or Graduate Degrees	2020	%	0.73	0.20	0.02	0.78	1.00
	Owner-occupied Housing Units	2020	%	0.60	0.32	0.00	0.67	1.00
	Ownership of Vehicle	2020	%	0.95	0.08	0.20	0.99	1.00

### 3.3 Methods

This study employed threshold analysis and equity toolkits to analyze EVCS access disparities in Austin. The majority threshold was defined as proportions greater than 50%. The research was divided into three phases. First, a cross-tabulation analysis was conducted based on the number of EVCS in each CBG. Three scenarios for EVCS access were tested: 1) Level 2 and Level 3 chargers; 2) only Level 2 chargers; and 3) only Level 3 chargers. We categorized each CBG by majority race and their proportions relative to the total number of CBGs. The race groups were Non-Hispanic White, Hispanic, and Black and Other race groups. The ‘Other’ group includes Asian, Native Hawaiian, Pacific Islander, Other, and Mixed Race. CBGs with a Black majority (greater than 50%) did not exist in Austin. CBGs with no majority race remained as a Reference group. However, omitting the Reference group would perpetuate structural segregation. To address this issue, the identified Reference groups underwent separate cross-tabulation analyses. Reference groups were further categorized based on the majority population within each, specifically into Black, American Indian, and Alaska Native, Asian, Native Hawaiian, and Pacific Islander, Other, and Two or More Races categories. The Reference groups predominantly comprised three neighborhood categories: Black, Asian, and Two or More Races. Income threshold was divided into four groups based on median income: low (<$62,490), lower-middle (<$87,874), upper-middle (<$111,419), and high (> = $111,419).

Second, we analyzed public EVCS access disparity by race and income with respect to multi-family proportion and distance to the nearest public EVCS. Race was first simply categorized between a binary of Non-Hispanic White and Non-White groups. We then divided the Non-White group into Hispanic and other major Reference groups (Black, Asian, and Two or More Races). The ’Other’ race category, previously defined based on the majority race, was excluded from this analysis due to its relatively small sample size compared to the other major Reference groups. Income group threshold categorization remained the same. The proportion of multi-family units and distance to the nearest public EVCS were rounded to specific thresholds to simplify the analysis. The axis of each threshold represents a value that is less than the following defined threshold. The rationale behind employing the proportion of multi-family units is due to the findings that residents in multi-family units need more optimum access to public chargers than residents in single-family units [11, 13]. Looking at the relative access to EVCS concerning distance to the nearest public EVCSs is to measure continuous effects.

The distance to the nearest public EVCS was spatially validated by conducting Anselin Local Moran’s I analysis. Anselin Local Moran’s I detects spatial groupings of observations that exhibit either high or low values. Having a positive value for the Moran’s I index indicates that neighboring features have similarly high or low attribute values. Clusters form with either consistently high values (high-high) or consistently low values (low-low). It also identifies outliers. A high value surrounded by low values is described as a high-low outlier. Conversely, a low value surrounded by high values is referred to as a low-high outlier. We conducted the Anselin Local Moran’s I test three times: (1) distance to the nearest Level 2 public EVCS, (2) distance to the nearest DC Fast Charging station, and (3) distance to the nearest Level 2 or DC Fast Charging station. Anselin Local Moran’s I follows the equation below [61]:

$$I_{i}=\frac{x_{i}-\bar{X}}{S_{i}^{2}}\sum_{j=1,j\neq i}^{n}\;w_{ij}\left(x_{i}-\bar{X}\right)$$ (1)

Where *x*_*i*_ is an attribute for observation *i*; $\bar{X}$ is the mean of the corresponding attribute; *w*_*ij*_ is the spatial weight between features *i* and *j*:

$$S_{i}^{2}=\frac{\sum_{j=1,j\neq i}^{n}{\left(x_{i}-\bar{X}\right)}^{2}}{n-1}$$ (2)

With *n* equating to the total number of observations, and *z*_*Ii*_-score for the statistics is computed using:

$$z_{Ii}=\;\frac{I_{i}-\text{E}\left[I_{i}\right]}{\sqrt{\text{V}\left[I_{i}\right]}}$$ (3)

Where:

$$\text{E}\left[I_{i}\right]=\;-\frac{\sum_{j=1,j\neq i}^{n}\;w_{ij}}{n-1}$$ (4)

$$\text{V}\left[I_{i}\right]=\text{E}\left[I_{i}^{2}\right]-\text{E}{\left[I_{i}\right]}^{2}$$ (5)

Third, after verifying non-linear relationships based on specific threshold definitions, we conducted a Generalized Additive Model (GAM) regression analysis to visualize the partial effects of explanatory variables on the dependent variable. GAM was chosen over the simple Ordinary Least Squares (OLS) model due to its effectiveness in visualizing non-linear relationships by replacing the beta coefficient with spline functions. We used a GAM regression with the assumption of a Gaussian distribution. Our study assumed that the likelihood of having public EVCS installed depends on a set of collected independent variables with relationships that are smooth but not necessarily linear. The Chi-Squared test was also significant (p<0.001) in the decision to use GAM over OLS. The GAM regression model had lower AIC, GCV, and higher adjusted R^2^ than OLS, as summarized in Table 2. The MGCV package in R was used for this analysis [62], and pairwise concurvity tests revealed that the independent variables had a concurvity value of less than 0.8 in the GAM.

**Table 2:** Comparison between OLS and GAM.

Model	AIC	GCV	Adjusted R^2^
OLS	-926.95	3.93e-05	0.31 (R^2^: 0.37)
GAM	-984.45	2.82e-05	0.63 (R^2^: 0.75)

When working with spatial data, it is important to consider spatial dependency [63]. This contradicts a fundamental assumption in linear regression models, which assumes that observations are independent of each other. However, GAMs are inherently flexible, which enables them to model complex, non-linear relationships between the dependent variable and one or more predictors. This flexibility comes from the use of smooth functions to model relationships, allowing the data to reflect the relationship’s form rather than imposing a strict linear or parametric structure [64]. Despite their flexibility, GAMs retain a level of interpretability. GAM does not impose any assumptions about the parametric relationships among variables [65]. GAMs offer the advantage of flexibility in modeling non-linear relationships, which can improve the fit of the model and potentially reduce prediction error compared to strictly parametric models [65]. When addressing challenges posed by spatial autocorrelation, GAMs can be extended to include spatial terms or smooths that can help account for spatial structure in the data. The foundational study by Hsu and Fingerman [13] also used GAM. Analyzing non-linear relationships with GAMS would enhance our understanding of the factors influencing access to public EVCSs and provide a more accurate quantification of existing disparities. GAM with a Gaussian error distribution is described by Hastie et al. [65], following the equation below.

$$Y_{i}=\beta_{0}+\sum_{j=1}^{n}\;s_{j}\left(x_{ij}\right)+\varepsilon_{i}$$

Where *Y*_*i*_ is the *i*th observation; *β*_0_ is the intercept of the GAM regression model; *s*_*j*_(*x*_*ij*_) is the *j*th smoothing function of covariate *j* for the *i*th sample; *ε*_*i*_ is the *i*th residual.

Each independent variable entered in our GAM model represents different socioeconomic and geographical factors affecting the likelihood of having EVCS installed. We built an equivalent model and tested several GAM models constructed by combining different independent variables. A log-likelihood test was performed to compare these models. The model that provided the best explanation was chosen as the optimal model. Our GAM regression model uses the following equation below.

$$\begin{array}{ll} EVCS_{k} & =\beta_{0}+\beta_{1}\cdot UrbanDummy_{k}+s_{1}\left(Non-HispanicWhite_{k}\right)\\ & +s_{2}\left(NumberofRegisteredEV_{k}\right)+s_{3}\left(MedianHouseholdIncome_{k}\right)\\ & +s_{4}\left(ResidentsAbovePoverty_{k}\right)+s_{5}\left(NearestDistanceEVCS_{k}\right)\\ & +s_{6}\left(NearestDistanceHighways_{k}\right)+s_{7}\left(MultiFamilyHousingUnits_{k}\right)\\ & +s_{8}\left(Education_{k}\right)+s_{9}\left(OwnerOccupiedHousingUnits_{k}\right)\\ & +s_{10}\left(VehicleOwnership_{k}\right)+\;\varepsilon_{k} \end{array}$$

Where *EVCS*_*k*_ is the likelihood of having EVCS at the corresponding CBG *k; β*_0_ is the intercept of the GAM regression model; *β*_1_ the coefficient for the UrbanDummy variable; *s*_*j*_(⋅) is the *j*th smoothing function for the corresponding covariate; *ε*_*k*_ is the residual for CBG *k*.

Lastly, we calculated the estimated values of the dependent variable across classified groups based on race and income using the finalized GAM regression model. We considered samples from major racial groups, including Non-Hispanic White, Hispanic, Other, and the Reference group. For income, we sampled and used four quartile groups: low, lower-middle, higher-middle, and high for the estimation. We took the average of each group’s observations for computation purposes.

## 4. Results

### 4.1. Descriptive statistics

If we calculate the ratio of EVs to public EVCS by CBGs, one charger is responsible for handling approximately three EVs on average in Austin. However, in some regions, the ratio goes beyond covering more than a hundred EVs.

Descriptive statistics of the study area indicate that of the 1206 CBGs in our study area, there were 674 White, 194 Hispanic, 12 Other, and 326 Reference groups. Based on median household income in CBG, there were 301 low, 302 lower-middle, 302 higher-middle, and 301 high income groups. Tables 3–5 summarize the cross-tabulation results, which show the proportion of observations for each group. These indicate that EVCS were installed in a relatively small number of CBGs, but the majority of White CBGs were more likely to have one or more EVCS compared to other races (see Table 3A). This disparity persists, even when categorizing them specifically into Level 2 chargers or Level 3 DC fast chargers (see Table 3B and 3C). As stated previously, Reference groups represent Black, Asian, or Two or More Races in a CBG. The majority of chargers Level 2 or Level 3 installed in these CBGs were mainly installed in Black communities (see Table 4).

**Table 3:** 

**A. Cross-tabulation between the total number of EVCSs and Race Groups**				
	Total Number of EVCS (Level 2 & 3)			

Race Group	None	One	Two	Three or More
Non-Hispanic White	555 (55%)	57 (63%)	27 (61%)	35 (51%)
Hispanic	166 (17%)	11 (12%)	6 (14%)	11 (16%)
Other	11 (1%)	0 (0%)	0 (0%)	1 (1%)
Reference Group	271 (27%)	22 (24%)	11 (25%)	22 (32%)
Total % in Row	1003 (100%)	90 (100%)	44 (100%)	69 (100%)
**B. Cross-tabulation between the Number of Level 2 EVCSs and Race Groups**				
	Number of Level 2 EVCS			

Race Group	None	One	Two	Three or More
Non-Hispanic White	561 (55%)	54 (63%)	28 (62%)	31 (49%)
Hispanic	168 (17%)	9 (10%)	6 (13%)	11 (17%)
Other	11 (1%)	0 (0%)	0 (0%)	1 (2%)
Reference Group	272 (27%)	23 (27%)	11 (24%)	20 (32%)
Total % in Row	1012 (100%)	86 (100%)	45 (100%)	63 (100%)
**C. Cross-tabulation between the Number of Level 3 DC Fast Charging Stations and Race Groups**				
	Number of Level 3 DC Fast Charging Station			

Race Group	None	One	Two	Three or More
Non-Hispanic White	654 (56%)	14 (64%)	1 (33%)	5 (83%)
Hispanic	190 (16%)	3 (14%)	1 (33%)	0 (0%)
Other	12 (1%)	0 (0%)	0 (0%)	0 (0%)
Reference Group	319 (27%)	5 (33%)	1 (33%)	1 (17%)
Total % in Row	1175 (100%)	22 (100%)	3 (100%)	6 (12%)

**Table 4:** 

**A. Cross-tabulation between the Total Number of EVCSs and Reference Race Groups**				
	Total Number of EVCS (Level 2 & 3)			

Reference Race Group	None	One	Two	Three or More
Non-Hispanic Black	189 (70%)	15 (68%)	7 (64%)	14 (64%)
Non-Hispanic Asian	67 (25%)	7 (32%)	3 (27%)	8 (36%)
Non-Hispanic Two or More Races	15 (6%)	0 (0%)	1 (9%)	0 (0%)
Total % in Row	271 (100%)	22 (100%)	11 (100%)	22 (100%)
**B. Cross-tabulation between the Number of Level 2 EVCSs and Reference Race Groups**				
	Number of Level 2 EVCS			

Reference Race Group	None	One	Two	Three or More
Non-Hispanic Black	189 (69%)	17 (74%)	7 (64%)	12 (60%)
Non-Hispanic Asian	68 (25%)	6 (26%)	3 (27%)	8 (40%)
Non-Hispanic Two or More Races	15 (6%)	0 (0%)	1 (9%)	0 (0%)
Total % in Row	272 (100%)	23 (100%)	11 (100%)	20 (100%)
**C. Cross-tabulation between the Number of Level 3 DC Fast Charging Stations and Reference Race Groups**				
	Number of Level 3 DC Fast Charging Station			

Reference Race Group	None	One	Two	Three or More
Non-Hispanic Black	221 (69%)	2 (40%)	1 (100%)	1 (100%)
Non-Hispanic Asian	82 (26%)	3 (60%)	0 (0%)	0 (0%)
Non-Hispanic Two or More Races	16 (5%)	0 (0%)	0 (0%)	0 (0%)
Total % in Row	319 (100%)	5 (100%)	1 (100%)	1 (100%)

**Table 5:** 

**A. Cross-tabulation between the Total Number of EVCSs and Income Quartiles**				
	Total Number of EVCS (Level 2 & 3)			

Income Group	None	One	Two	Three or More
Low (<$62,490)	242 (24%)	24 (27%)	16 (36%)	19 (28%)
Lower-middle (<$87,874)	243 (24%)	29 (32%)	10 (23%)	20 (29%)
Higher-middle (<$111,419)	250 (25%)	23 (26%)	10 (23%)	19 (28%)
High (> = $111,419)	268 (27%)	14 (16%)	8 (18%)	11 (16%)
Total % in Row	1003 (100%)	90 (100%)	44 (100%)	69 (100%)
**B. Cross-tabulation between the Number of Level 2 EVCSs and Income Quartiles**				
	Number of Level 2 EVCS			

Income Group	None	One	Two	Three or More
Low (<$62,490)	243 (24%)	24 (28%)	17 (38%)	17 (27%)
Lower-middle (<$87,874)	249 (25%)	25 (29%)	9 (20%)	19 (30%)
Higher-middle (<$111,419)	251 (25%)	23 (27%)	10 (22%)	18 (29%)
High (> = $111,419)	269 (27%)	14 (16%)	9 (20%)	9 (14%)
Total % in Row	1012 (100%)	86 (100%)	45 (100%)	63 (100%)
**C. Cross-tabulation between the Number of Level 3 DC Fast Charging Stations and Income Quartiles**				
	Number of Level 3: DC Fast Charging Station			

Income Group	None	One	Two	Three or More
Low (<$62,490)	296 (25%)	3 (14%)	1 (33%)	1 (17%)
Lower-middle (<$87,874)	290 (25%)	9 (41%)	1 (33%)	2 (33%)
Higher-middle (<$111,419)	294 (25%)	6 (27%)	1 (33%)	1 (17%)
High (> = $111,419)	295 (25%)	4 (18%)	0 (0%)	2 (33%)
Total % in Row	1175 (100%)	22 (100%)	3 (100%)	6 (100%)

For income, within the CBGs with EVCSs, lower and lower-middle incomes were slightly more likely to have both Level 2 and Level 3 EVCSs than the other income groups (see Table 5A). This trend is also reflected in the distribution of only Level 2 chargers (see Table 5B). On the other hand, Level 3 DC fast chargers were predominantly installed in lower-middle-income regions (see Table 5C). However, higher-middle-income groups are more likely than low-income groups to have DC fast chargers installed in their areas. The wealthiest neighborhoods also have a higher likelihood of having DC fast chargers compared to the low-income groups, with the exception of the probability of having two DC fast chargers installed.

Table 6 presents the results of cross-tabulating race and income quartiles. Across the income categories, the White alone population constitutes the majority of CBGs, except for the low-income quartile (see Table 6A). The majority of the low-income quartiles are comprised of Hispanic and other Reference groups. The Reference groups were predominantly Black in the low to higher-middle-income categories, and predominantly Asian for the highest income group (see Table 6B).

**Table 6:** 

**A. Cross-tabulation between Race Groups and Income Quartiles**				
	Income Quartiles			

Race Group	Low	Lower Middle	Higher Middle	High
Non-Hispanic White	82 (27%)	154 (51%)	183 (61%)	255 (85%)
Hispanic	112 (37%)	46 (15%)	36 (12%)	0 (0%)
Other	1 (0%)	1 (0%)	2 (1%)	8 (3%)
Reference Group	106 (35%)	101 (33%)	81 (27%)	38 (13%)
Total % in Row	301 (100%)	302 (100%)	302 (100%)	301 (100%)
**B. Cross-tabulation between Reference Race Groups and Income Quartiles**				
	Income Quartiles			

Reference Race Group	Low	Lower Middle	Higher Middle	High
Non-Hispanic Black	83 (78%)	71 (70%)	54 (67%)	17 (45%)
Non-Hispanic Asian	16 (15%)	26 (26%)	24 (30%)	19 (50%)
Non-Hispanic Two or More Races	7 (7%)	4 (4%)	3 (4%)	2 (5%)
Total % in Row	106 (100%)	101 (100%)	81 (100%)	38 (100%)

### 4.2. EVCS access disparity in race and income

**Fig 2:**
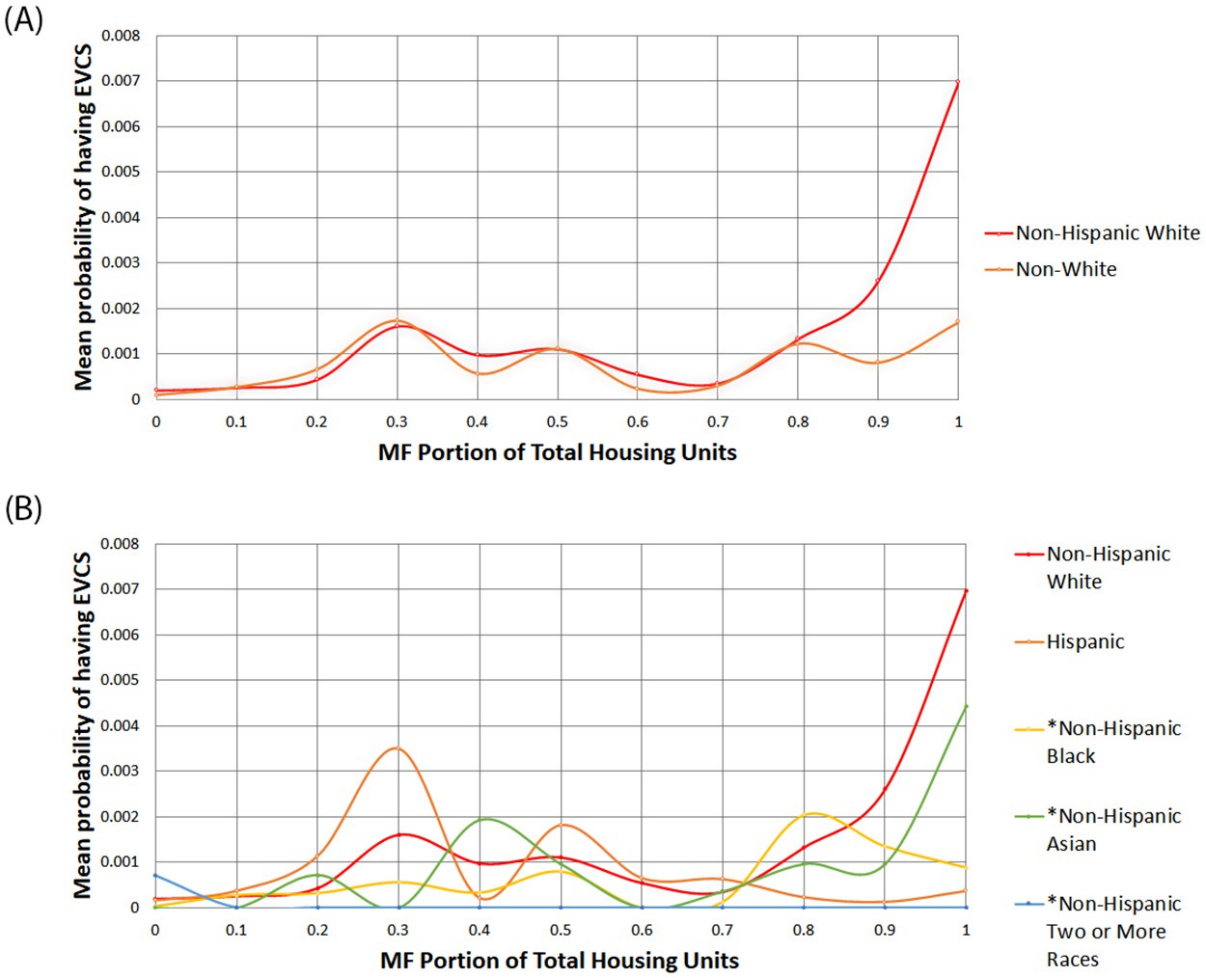
**A.** Comparison of public EVCS access between simplified race groups by multi-family portion. **B.** Comparison of public EVCS access between multiple race groups by multi-family portion.

**Fig 3:**
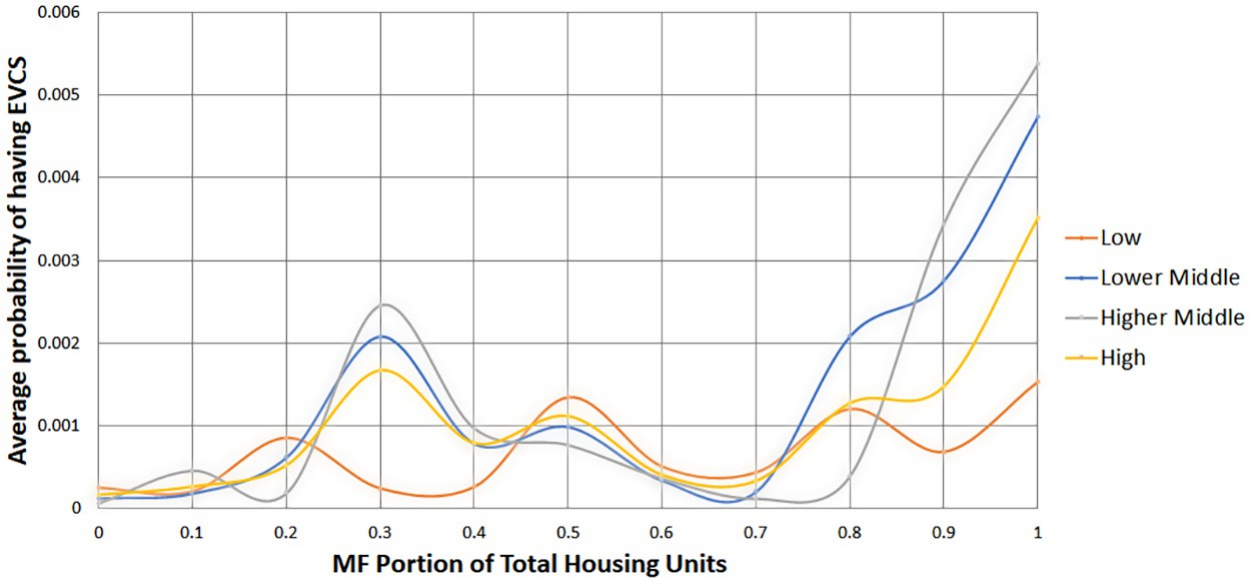
Comparison of public EVCS access between income quartiles by multi-family portion.

Figs 2 and 3 compare public EVCS access between the race and income quartile groups compared to the proportion of multi-family units. ‘*’ indicates a Reference group with a total of 326 Reference groups. The results indicate that the Non-Hispanic White group was more likely than the Non-White group to have access to a nearby EVCS in areas with a multi-family ratio of 80% or more (see Fig 2A). When the Non-White group is divided into Hispanic and Reference groups (which include Black, Asian, and individuals of Two or More Races), their probabilities fluctuate (see Fig 2B). At the point where the multi-family ratio is 30%, the Hispanic population is most likely to have access to chargers. However, the disparity remains even when the multi-family ratio reaches 80%. Generally, the White alone population has the highest likelihood of having charger access, followed by the Black and Asian populations. In contrast, the Hispanic and mixed-race groups have the least likelihood, with their chances nearly approaching zero. Using the 80% multi-family threshold, middle- and high-income groups had a higher chance of having access to a nearby EVCS than low-income groups (see Fig 3). It should be noted that the higher-middle and lower-middle classes had greater access than the high-income group.

**Fig 4:**
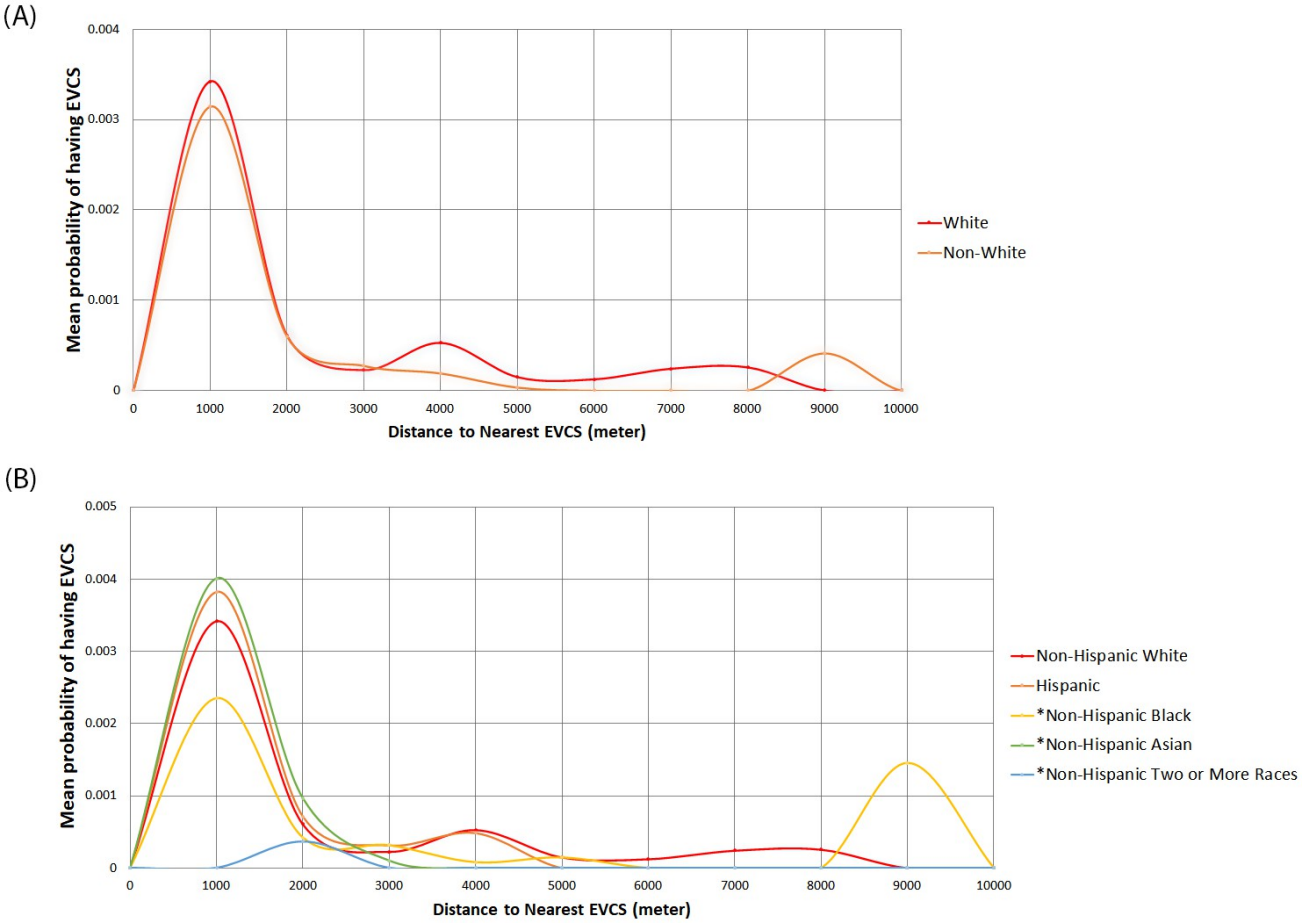
**A.** Simplified comparison of public EVCS access between race groups by distance to nearest public EVCS. **B.** Comparison of public EVCS access between multiple race groups by distance to the nearest public EVCS.

**Fig 5:**
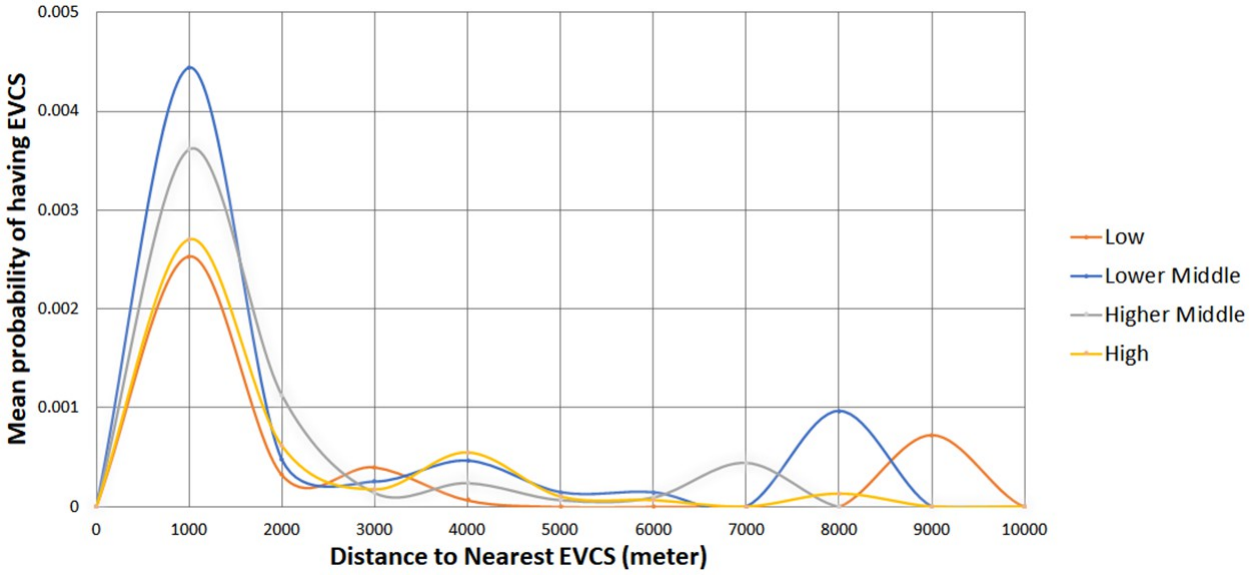
Comparison of public EVCS access between income quartiles across distance to the nearest public EVCS.

Figs 4 and 5 compare public EVCS access between the race and income quartile groups compared to the distance to the nearest public EVCS. Again, ‘*’ indicates a Reference group with a total of 326 Reference groups. The results indicate that Non-Hispanic White groups living less than 2 km from public EVCS have a higher mean probability of having access to a nearby public EVCS than Non-White groups (see Fig 4A). The disparity is also evident when comparing the Black or mixed-race population with the White alone population (see Fig 4B). However, the Asian and Hispanic populations, when living within 2 km of public EVCSs, show a greater likelihood of having access to chargers. Middle-income and high-income groups living less than 2km have a greater probability of having EVCS than the low-income groups living at the same distance (see Fig 5). After 2 km, there is a marginal difference between the race and income groups, except for Black populations, which are, on average, about 9 km away from the nearest charger (see Fig 4B).

**Fig 6:**
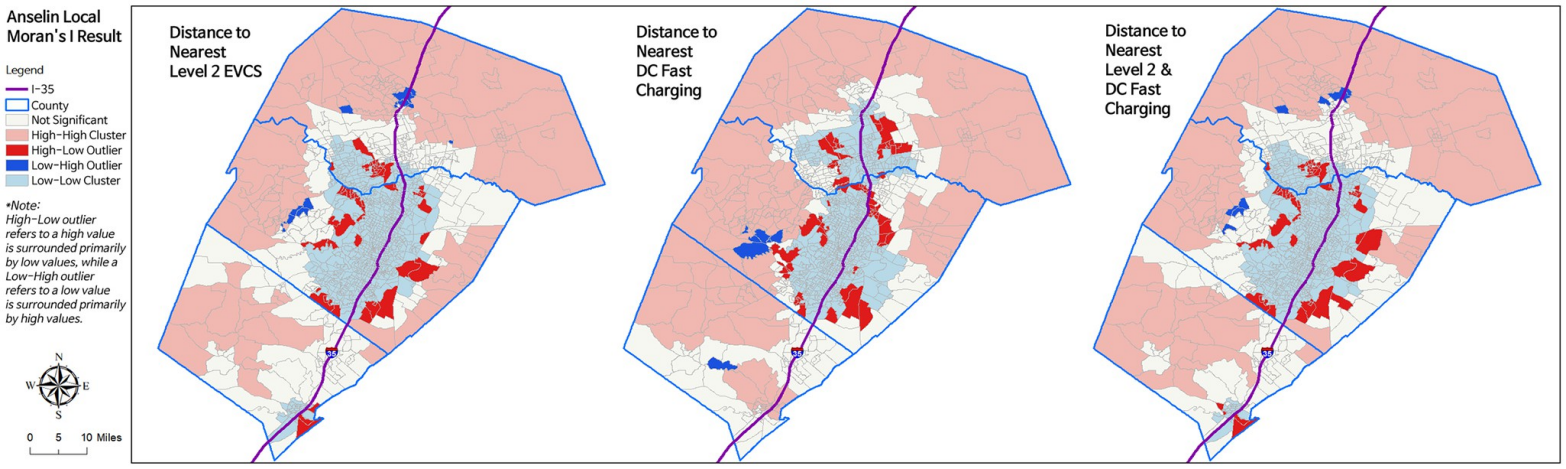
Anselin Local Moran’s I results for the nearest distance to the public EVCS.

Fig 6 illustrates Anselin Local Moran’s I for the nearest distance to the public EVCS. Central Austin, primarily located near public EVCS (either Level 2 or Level 3 DC fast charging), is identified as a low-low cluster by Moran’s I. Outskirts show clusters of high-high values, indicating they are located further from the nearest public EVCS. Some regions in West Austin and near I-35 are found to be closer to public EVCS, while their neighboring regions are further away. There are eight regions identified as low-high outliers by Moran’s I analysis for the overall nearest distance to public EVCS. All of these regions are White neighborhoods. Among these regions, 2 belong to high-income groups, 4 to upper-middle-income groups, and 2 to lower-middle-income groups.

Despite Central Austin having a relatively short distance to the public EVCS, some regions were relatively farther away and identified as high-low outliers in both West and East Austin. There are relatively more high-low outliers than low-high outliers. When conducting Moran’s I analysis for the overall nearest distance to the public EVCS, 52 high-low outliers were identified. Among them, 25 are White, 9 are Hispanic, 15 are Reference (10 are Asian and 5 are Black), and 3 belong to Other groups. We previously reported 674 White, 194 Hispanic, 12 Other, and 326 reference groups. Relatively speaking, 3.8% of Whites, 4.6% of Hispanics, 4.6% of Reference groups, and 25% of Other groups are located further from the public EVCS while their neighboring regions are in close proximity. The income group classification of these outliers includes 7 from low-income groups, 10 from lower-middle-income groups, 9 from higher-middle-income groups, and 26 from high-income groups. There were 301 low-income, 302 lower-middle-income, 302 higher-middle-income, and 301 high-income groups. Of these, 2.3% of low, 3.3% of lower-middle, 2.9% of higher-middle, and 8.6% of high-income groups are located further from public EVCS while their neighboring regions are in close proximity.

### 4.3. Generalized Additive Model Regression (GAM) analysis results

The results of the GAM regression are summarized in Table 7. Non-Hispanic White ratio (p<0.01), registered EV (p<0.01), median household income (p<0.001), poverty level: residents above poverty income level (p<0.01), distance to nearest public EVCS (p<0.001), and urban dummy variable (p<0.001) were statistically significant.

**Table 7:** Generalized additive model result^1^.

GAM Model								
Index	Estimate	Std. Error	t value	P-value	EDF	Ref.df	F-value	P-value
Constant	0.002	0.000	8.983	0.000***	--	--	--	--
Urban	0.002	0.000	8.983	0.000***	--	--	--	--
s(Non-Hispanic White)	--	--	--	--	7.922	8.656	2.922	0.004**
s(Registered EV)	--	--	--	--	5.294	6.247	3.701	0.002**
s(Median household income)	--	--	--	--	4.908	5.934	5.597	0.000***
s(Poverty level)	--	--	--	--	2.589	3.536	4.285	0.005**
s(Distance to nearest public EVCS)	--	--	--	--	8.705	8.959	6.195	0.000***
s(Distance to nearest highway)	--	--	--	--	2.138	2.472	2.305	0.075
s(Multi-family)	--	--	--	--	1.000	1.000	3.691	0.058
s(Education)	--	--	--	--	4.460	5.476	1.580	0.166
s(Owner occupancy)	--	--	--	--	1.000	1.000	1.546	0.217
s(Vehicle occupancy)	--	--	--	--	1.000	1.000	1.844	0.178

^1^*** p < 0.001; ** p < 0.01; *p < 0.05; s() indicates that the term is considered a non-linear term; Chi-Squared test (simple OLS < GAM***). The dependent variable is a proportion of EVCS in a particular CBG relative to all the CBGs.

The coefficient of the urban dummy variable represents the change in the dependent variable (e.g., having a public EVCS installed) for CBGs classified as urban with respect to those classified as rural. The coefficient for the urban dummy variable is 0.002, indicating that, holding all other variables constant, a one-unit increase in the urban dummy variable increases the probability of having a public EVCS installed by 0.2% for CBGs classified as urban compared to those classified as rural. For the smooth terms, we interpret their effects qualitatively by visualizing them, due to their nonlinear nature. The significance of these terms indicates that the Non-Hispanic White ratio, the number of registered EVs, median household income, poverty level, and distance to the nearest public EVCS have a substantial non-linear impact on the dependent variable.

**Fig 7:**
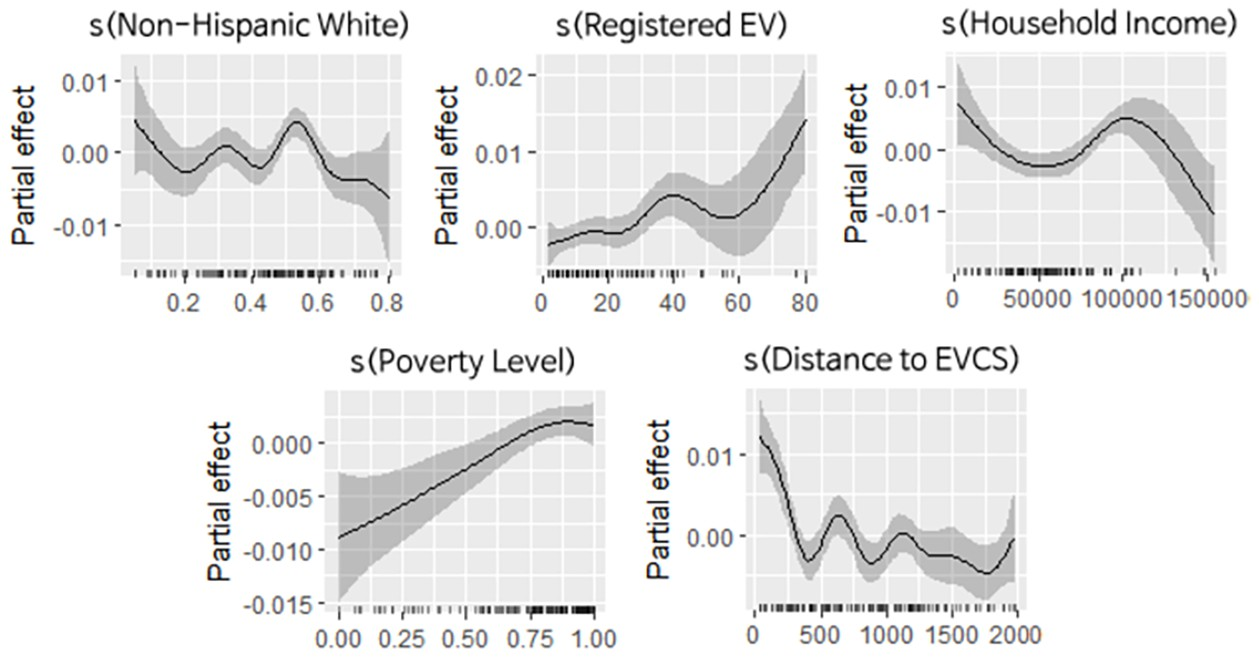
Estimated smoothness of statistically significant explanatory variables.

The partial effects of statistically significant non-linear predictors are illustrated in Fig 7. The X-axis represents the observation. The Y-axis refers to the partial effect of the variable. The shaded area represents a 95% confidence interval. The results indicate that registered EVs and the proportion of residents above the poverty income level have a positive impact on the dependent. Distance to the nearest public EVCS generally has a negative impact on the dependent. Median household income has a negative partial effect; however, positive partial effects are seen near the lower-middle income quartile range. At certain thresholds, the Non-Hispanic White ratio has inconsistent positive impacts on the dependent.

The estimation was conducted for classified groups based on race and income using a defined GAM regression model. For race, the average predicted probability of having an EVCS installed in CBGs was highest in the Non-Hispanic White group. The Non-Hispanic White group had an average predicted probability of 0.59%, the Hispanic group had -0.009%, the Other group had 0.34%, and the Reference group had 0.23%. In terms of income, the low-income group had an average predicted probability of 0.14%, the lower-middle-income group had 0.59%, the higher-middle-income group had 0.53%, and the high-income group had the highest average predicted probability at 1.27%.

## 5. Discussion

This study analyzed the public EVCS access disparity in Austin, TX, and aimed to extend transportation equity concerns to EV charging infrastructure. We based our research on studies of explicit charging equity, which are grounded in distributional philosophies. Our study employed a threshold analysis to categorize majority race and income groups and examine how existing public EVCSs are distributed across Austin. The public EVCS access disparity was later visualized to compare the continuous relationship between the proportion of multi-family units and the distance to the nearest public EVCS. The distance to the nearest public EVCS is further validated by conducting an Anselin Local Moran’s I analysis. Finally, our research conducted a GAM regression analysis to look at the relative impacts of possible determinants on public EVCS access. We conducted the estimation using a defined GAM regression model.

The key findings of this study are as follows. First, cross-tabulation between the number of public EVCSs and race groups showed that areas with a majority Non-Hispanic White population have a higher chance of accessing one or more public EVCSs than Non-White majority areas, with the majority of existing public EVCSs installed in regions where the majority population is Non-Hispanic White (either Level 2 or 3; see Table 3a–3c). When comparing public EVCS access disparity between proportion of multi-family units and distance to the nearest public EVCS, Non-Hispanic White groups had higher chance of accessing public EVCSs than Non-White groups based on our thresholds (see Fig 2A). The disparity is more significant in regions with a higher proportion of multi-family units (see Fig 2A). Since ensuring access to public EVCSs for residents in multi-family households is a key factor in transportation electrification equity [11], this access disparity should be addressed.

The Non-Hispanic White group generally had higher public EVCS access than Non-White groups based on the distance to the nearest public EVCS, with the disparity being most pronounced within distances of 2 km (see Fig 4A). Notably, low-high outliers identified in Moran’s I analysis, which are relatively closer to public EVCS compared to neighboring regions, were all classified as Non-Hispanic White neighborhoods (see Fig 6). Relatively speaking, the high-low outliers identified in Moran’s I analysis were more prevalent in Hispanic, Reference, and Other groups than in the White community (see Fig 6).

In areas characterized by a high prevalence of multi-family units (where multi-family residences constitute 80% of total housing units), the Non-Hispanic White population typically had the highest probability of accessing pubic EVCSs, compared to Hispanic, Asian, Black, and mixed-race groups (see Fig 2B). Interestingly, when considering populations within the Reference group, Asian and Hispanic communities had a higher likelihood of living in proximity to public EVCSs compared to White, Black, and mixed-race groups (see Fig 2B). However, it should be noted that Black and mixed-race communities are consistently underrepresented in this context (see Fig 2B).

Second, the deployment of public EVCSs is more equally distributed across income in Austin based on cross-tabulation results between public EVCS and income quartiles (see Table 5A–5C). However, when comparing public EVCS access between income quartiles between the proportion of multi-family units and distance to the nearest public EVCS, low-income groups had less access to public EVCSs in regions where more than 90% of housing units were multi-family (see Fig 3) or areas located less than 2 km from the nearest public EVCS (see Fig 5). These results support the argument that installing more chargers without considering regional characteristics would reinforce preexisting structural disparities. High-income groups having less access than median income groups in the same threshold are likely due to their having more access to opportunities to charge their EVs without using a public EVCS. For example, the multi-family units they live in may possess private EVCS, or they may have personal garages to install at-home EVCS and are less reliant on living near public EVCS than median income groups. A similar result is confirmed in Moran’s I analysis, where high-income communities had a 2–3 times higher chance of being high-low outliers for the nearest distance to public EVCS compared to other income quartile groups (see Fig 6). Even so, high-income groups had greater access to the low-income groups in regions where more than 80% of housing units were multi-family or regions located less than 2 km from the nearest public EVCS. The low-income community did not appear as a low-high outlier for the nearest distance to public EVCS in Moran’s I analysis (see Fig 6).

The cross-tabulation of race and income quartiles indicates that the low-income group primarily comprises the Hispanic and Reference groups, with the Hispanic community representing the largest portion of the low-income group (see Table 6B). Within the Reference group, the Black community is the most prevalent (see Table 4C). Prioritizing the needs of the low-income groups due to their limited access to chargers should entail advocating for the needs of low-income Hispanic and low-income Black populations in Austin.

Third, our GAM regression result showed that regional and socio-demographic characteristics have a statistically significant impact on public EVCS access disparity (see Table 7 and Fig 7). Incorporating the GAM in our analysis was crucial to elucidating non-linear relationships, which is a common and significant feature in socio-demographic and transportation data [66–68]. Drawing from the foundational study by Hsu and Fingerman [13], unlike linear models that assume a constant rate of change, GAMs adeptly capture the varying influence of socio-economic and urban factors on public EVCS accessibility. This allowed us to identify intricate patterns that linear models might overlook. As such, GAM enables us to pinpoint specific socio-demographic thresholds or tipping points beyond which the availability of EVCS markedly increases or decreases.

One notable characteristic is the disparity between urban and rural regions. Since urban areas tend to have more EV registrations and a proportion of residents above the poverty level, public EVCS access would significantly increase. This result indicates that Austin follows the trend of early installation of public EVCSs being skewed towards urban areas [2–4]. Another notable characteristic is the disparity between races in areas with a Non-White majority. There are cases where the proportion of Non-Hispanic White positively impacts public EVCS access up to the threshold where Non-Hispanic White increase above the majority, where the impact is flipped. A similar result is seen when examining the increasing median household income. The estimation showed that the Non-Hispanic White group had a greater chance of having public EVCS installed than other racial groups. The Hispanic group had the least access, with a negative likelihood estimation. The high-income group had the highest chance of having public EVCS installed compared to other income groups. Compared to the low-income group, the high-income group had a nine-times higher likelihood (0.14% compared to 1.27%) of having public EVCS installed.

Our study adopted a comprehensive approach to identify disparities in charger access. Previous research acknowledged that different demographic groups require prioritization due to charging disparities, notably between income groups. Past studies like Canepa et al. [3] focused on income, while others like Hsu and Fingerman [13], Carlton and Sultana [15], Khan et al. [7], and Min et al. [16] incorporated race or location (urban vs. rural). Our analysis took a more comprehensive approach and offered a nuanced perspective by categorizing race more granularly and considering multiple major factors that can influence disparities in EV charger access, such as race, income, and location. This pluralistic approach in our study provides a more detailed understanding of spatial inequalities in EV charger adoption.

Furthermore, we highlight the importance of broadening the scope of research on public EVCS access beyond the West Coast, emphasizing the need to explicitly incorporate equity and justice considerations within the domain of transportation equity research. Pragmatically speaking, it’s crucial for research to evolve continuously, addressing disparities effectively by continuously being questioned [69]. Our work contributes to this evolving discourse by conducting a case study in Austin. We enrich the conversation around charging equity with tangible insights and reinforce the need for normative standards in such studies.

Borrowing the research framework from energy justice, energy planning and policy, externalities, and impacts on disadvantaged groups, along with recognizing the benefits and burdens [70], needs to be further identified to locate the cause of the existing condition. Locating where injustices occur and where energy is consumed and purchased [71] would contribute to understanding the power distribution status. Low-income communities have lower access to smart grid technology [72]. Therefore, disadvantaged communities that have less access to public EVCS may find it fundamentally less feasible to install public EVCS nearby due to spare capacity. Getting real-time electricity use data by region or household is quite complicated in TX due to homeland security reasons. To analyze the root cause, collaboration with government entities and electricity operators would be necessary. Level 2 and Level 3 DC fast charging show different charging profiles [73]. Level 2 is used relatively more during the weekdays, with peak usage on office, medical, or educational campuses, while DC fast charging is highly used on weekends, with the highest number of uses per day at municipal buildings [73]. After the COVID-19 pandemic, the use of DC fast charging has increased [73]. DC fast charging is a more optimal solution for meeting long-distance travel demand than Level 2 charging [74] and is suitable for installation in major transportation corridors [75]. High-high clusters identified in our Moran’s I analysis and high-low outliers in proximity to I-35 (see Fig 6), identified as low-income or communities of color, might be suitable for additional DC fast charging installations. This could be a business opportunity for charging operators to expand their service regions. Here, the association between crime rates and the use of public spaces in low-income regions is reported [76]. Possible crime cases near public EVCS should be addressed to ensure their availability.

## 6. Conclusions

This research analyzed public EVCS access disparities in Austin, TX. As local and federal agencies work together to promote transportation electrification plans, the findings of this study may be used to identify existing equity issues with public EVCSs. The contribution of the studies is as follows.

First, our study contextualizes existing transportation equity and charging equity discourse to address public EVCS access concerns in Austin. Our pluralistic method, which disaggregates Reference groups and addresses factors in a comprehensive yet fragmentary manner, is widely applicable in future studies. Typically, charging equity studies adopt methods rooted in distributional philosophies, often employing a simple equality approach [12]. It is understood as a normative dialogue, recognizing that completely eliminating disparities might not be feasible. Consequently, a more pragmatic approach would be to concentrate on mitigating the issue. This shifts the focus from seeking absolute solutions to implementing practical strategies that reduce disparities in public EVCS access.

However, it’s important to note that quantifying issues partially doesn’t entirely resolve the problem. Thus, while distributional approaches are effective in investigating existing issues, they are not adequate for achieving mobility justice [45]. Purely distributional approaches address the issue only partially, making it challenging to align with political movements and effect change [45]. To overcome this limitation, we propose exploring how the capability approach can offer fresh perspectives on addressing charging equity issues. It’s also important to address how the results from equity studies can effectively facilitate communication with community advisory groups [77]. Karner et al. [45] recently compared a set of methods in transportation equity analysis when conceptualized with accessibility inequality based on distributional philosophy and accessibility poverty based on sufficientarian philosophy. As EVCS accessibility analysis in charging equity studies is mainly based on distributional philosophy [12], further studies are needed to uncover the limitations of the existing research framework and to improve practice with new methodological results.

Second, our research found that there were public EVCS access disparities in Austin, TX, across race and income. Using our thresholds, Non-Hispanic White groups and higher income groups had better access to public EVCSs than Non-White groups and low-income groups. The results follow equity issues found in existing studies [2–4, 7, 13]. Based on the cross-tabulation results, advocating for low-income groups necessitates a focus on the specific needs of low-income Hispanic and Black populations in Austin, especially those residing in multi-family housing units. While there are instances where Asian and Hispanic populations have greater access to nearby public EVCSs, access alone doesn’t guarantee the capability to utilize them. Furthermore, the data consistently shows that Black and mixed-race communities are underrepresented in terms of charger accessibility. Addressing these disparities requires targeted advocacy and policy interventions to ensure equitable access and usability of charging infrastructure for all communities.

Third, our GAM regression results found that communities with more registered EVs, lower poverty levels, closer proximity to public EVCSs, and located in urban areas have a better chance accessing public EVCSs. Comparing historically disadvantaged races and income groups, such as lower-income and minority communities, shows a more pronounced disparity. The estimation of our GAM regression model confirmed that the Non-Hispanic White group and high-income group had the highest access to public EVCS.

Policymakers in Austin have been working to expand public EVCS access to disadvantaged communities. Although they are valid in addressing differences across race and income, we encourage them to go beyond traditional threshold classifications and consider more concrete measures to address the needs of disadvantaged communities. For one, they could consider the proportion of multi-family units or the distance to the nearest public EVCS as other measures of equity. Moreover, they should improve the collection and publication of their data to accurately identify disadvantaged areas. Using community outreach programs and directly communicating with leaders in historically disadvantaged regions would improve both transparency and accuracy in addressing equity concerns. How smart grid technology and the promotion of smart meters ensure smart charging with regard to issues of energy justice is a valuable area for future research.

Following the White House’s Justice40 initiative, the U.S. Department of Transportation acknowledged the importance of ensuring access to affordable EVs and charging stations, leading to the creation of the Charging Justice40 Map tool project. However, our analysis reveals discrepancies in the provided data; the charging points do not fully represent the existing conditions, primarily because the tool only accounts for non-Tesla DC fast chargers. Moreover, the EV Corridor is limited to the I-35 highway. The concept of an EV grid encompasses more than just highways; it includes a variety of charging points, differing by type and operator. A comparative analysis with the existing vulnerability map in Austin could reveal disparities that may be overlooked by using a broader methodology. As the Justice40 initiative aims to allocate 40 percent of the benefits from climate, clean energy, affordable and sustainable housing, clean water, and other investments to disadvantaged communities, pinpointing isolated, underserved communities within this framework is valuable. It would ensure that the National EV Infrastructure Formula program’s funds and resources are directed more effectively towards the ‘disadvantaged’ communities. This could entail installing more EVCS in historically underserved areas, promoting job creation, workforce development, and training opportunities in the clean energy sector, such as jobs related to installation.

Lastly, it is important to note that this study was limited by relying on existing transportation equity frameworks and did not explore the broader issues of transportation justice. Our research framework heavily applies the principles of distributional philosophy. More computational studies are required using different conceptual frameworks of transportation equity and charging equity. Several assumptions were made to simplify the study’s methodology. Our data was limited by the public-private partnership nature of the EV market. Future studies would benefit from having better access to data such as the Level 1 charger installation rate, the operators running public EVCS, and quantified measurements of charging usage.

## Supporting information

S1 Table Current incentive and rebate program offered by Austin Energy. (DOCX)
