# Supplementary material for: Toward an equitable transportation electrification plan: Measuring public electric vehicle charging station access disparities in Austin, Texas
Source: PLoS One. 2024 Sep 5;19(9):e0309302. doi: 10.1371/journal.pone.0309302 (PMC11376518; doi:10.1371/journal.pone.0309302)
Supplement: S1 Table — (DOCX) [file pone.0309302.s001.docx]

**Appendix**

**S1 Table**. Current Incentive and Rebate Program Offered by Austin Energy

| **Charing Station Type** | | **Rebate Amount** |
| --- | --- | --- |
| Residential Charging:  Single-family  Home Charging  (Level 2 Plug-in) | | - Rebate of 50% and installation cost of an approved Level 2 - Maximum Rebate amount is $1,200 for Wi-Fi enabled option, $900 for non-Wi-Fi enabling option |
| Residential Charging:  Multifamily  Charging: Benefits for Property Owners | Independent EVCS Rebate | - Up to $700 per bank of Level 1 - Up to $1500 per OCPP-compliant station of Level 2 - Up to $1200 per-non OCPP station   *OCPP – Open Charge Point Protocol |
|  | Plug-In Austin Charging Station Rebate | - Up to $4,000 per station at existing construction - Up to $2,500 per station at new construction - Up to $15,000 per station |
| Commercial Charging | | - Same as Multi-family Property Owners |
| School Charging | | - Rebate of up to $4000 or 50% of the cost to install approved Level 2 or Level 1 outlets |
